# Electromagnetic field quantization and quantum optical input-output relation for grating

**DOI:** 10.1038/s41598-019-56197-1

**Published:** 2019-12-27

**Authors:** Tiecheng Wang

**Affiliations:** College of Physics and Electronic Engineering, Shanxi University, 030006 Taiyuan, China

**Keywords:** Quantum optics, Photonic crystals

## Abstract

A quantization scheme is developed for the radiation and higher order electromagnetic fields in one dimensional periodic, dispersive and absorbing dielectric medium. For this structure, the Green function is solved based on the plane wave expansion method, thus the photon operators, commutation relations and quantum Langevin equations are given and studied based on the Green function approach, moreover, the input-output relations are also derived. It is proved that this quantum theory can be reduced back to that of the predecessors’ study on the homogenous dielectric. Based on this method, we find that the transformation of the photon state through the lossy grating is non-unitary and that the notable non-unitary transformation can be obtained by tuning the imaginary part of the permittivity, we also discussed the excellent quantum optical properties for the grating which are similar to the classical optical phenomena. We believe our work is very beneficial for the control and regulation of the quantum light based on gratings.

## Introduction

In recent years, a significant effort has been devoted to the study on the theory and application of metastructure and metasurface, which are artificial periodic structures with their periodicity perpendicular to the incident direction of light^1^. Based on the dimension of the periodicity, they can be classified as one dimensional (1D) grating^2^ and two dimensional variation of such structures. A lot of fundamental study on these structures has been conducted, including band structure^3–5^, scattering^6,7^, absorption^8,9^, nonlinear optical effects^10–12^, and so on. The extraordinary features, like guided resonance, light bound states in the continuum (BICs)^13–15^, and so on, enable these structure to be applied to many optical processes, for example, hollow-core waveguide^16^, high-Q resonators^17^, surface-normal coupler^18^, vertical-cavity surface-emitting lasers^19^, high-NA planar lenses^20^, surface-normal second-harmonic emission^21^, and so on.

The previous study of metastructure and metasurface focuses on the classical optical properties and presents various and meaningful application in classical optics, but an important question is noticeable, how these structures regulate the quantum electromagnetic (EM) field? To solve this problem we should accomplish the the first two basic tasks which are the EM field quantization in those periodic artificial structures and getting the corresponding input-output relation. There has been extensive research on EM quantization^22–26^. In ref. ^27^, a fully canonical quantization scheme which is based on the Hopfield model^28^ of a dielectric for the macroscopic EM field in a linear harmonic oscillator bulk material is developed, the EM field is coupled to a harmonic-oscillator polarization field that interacts with a continuum of harmonic oscillator reservoir fields. Another method-the Green function approach-is introduced to solve the quantization problem for the dielectric including loss, which can be regarded as a natural extension of the familiar method of the mode expansion to arbitrary Kramers-Kronig consistent media. The quantization of the radiation field is based on the classical Green function representation of the vector potential, identifying the external sources therein with the noise sources that are necessarily associated with the loss in the medium. However, so far there has not been specific EM quantization theory for the medium with periodic structure.

In our work, the plane-wave expansion (PWE) method^29–31^, which is applied previously to deal with the classical optical problems for the periodic structures, and the Green function approach are used to accomplish the EM field quantization for 1D periodic, dispersive, and lossy medium. The EM fields in plane wave expansion is introduced to the quantum Maxwell equation and then the Green function in the corresponding bulk system can be calculated, on the basis of these the photon annihilators will be obtained. Moreover, we can study the input-output relation and get more quantum properties by applying this relation. Here the method we used is developed from the mode expansion of photon operators^32–34^.

## Theory and Method

### Solution of quantum Maxwell equations for 1D grating

**Figure 1:**
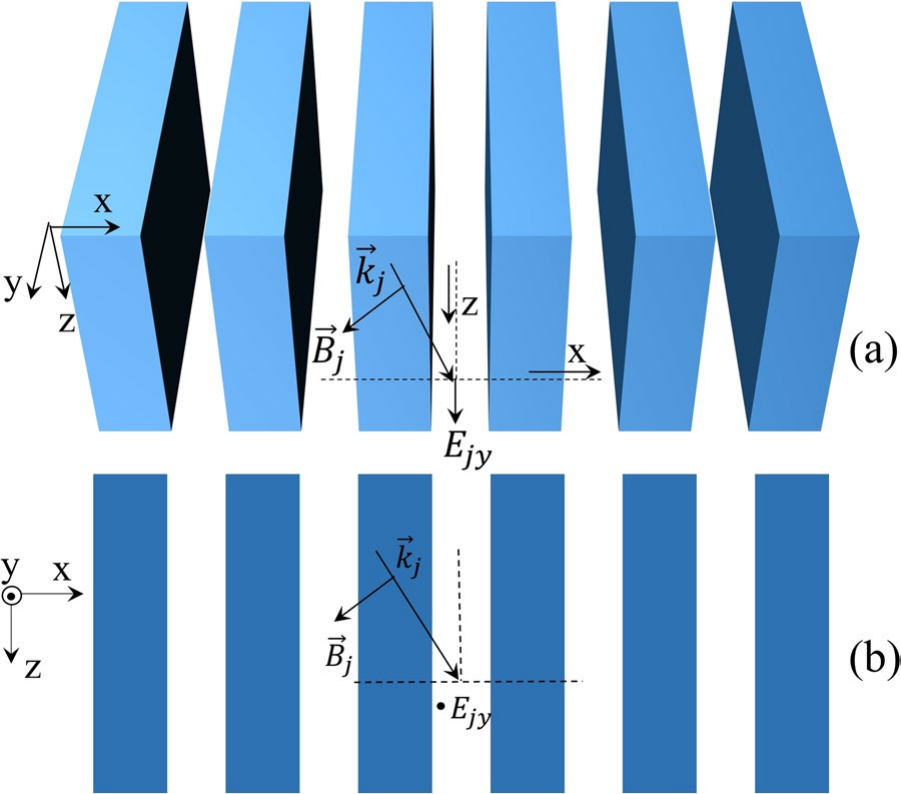
Monochromatic TE-polarized EM wave propagating in the 1D periodic medium shown in (**a**) (oblique view) and (**b**) (side view). The structure is periodic along x direction with period a, the wave vector lies in the x-z plane and the nonzero electric field is *E*_*y*_.

We consider 1D periodic structure (1D photonic crystal) as shown in Fig. 1. In order to quantize the eletromagnetic field in this structure we will resolve the quantum Maxwell equations^22–25^ using PWE method. Here the relative permittivity is periodic along the x direction and uniform along the y and z direction, we consider the transverse electric (TE) modes in which the electric field is polarized in the uniform y direction, all possible nonzero EM fields are denoted by $({\hat{H}}_{x},{\hat{E}}_{y},{\hat{H}}_{z})$. The unit vectors of the primitive lattice and the corresponding reciprocal lattice can be introduced ***a*** = *a****e***_*x*_ and ${\boldsymbol{b}}=b{{\boldsymbol{e}}}_{x}=2\pi /a{\overrightarrow{{\boldsymbol{e}}}}_{x}$, respectively. The operator EM fields can be written as the superposition of plane waves based on the PWE method

1a $${\hat{E}}_{y}(x,y,z,\omega )=\sum _{j}\,{\hat{E}}_{jy}(z,\omega ){e}^{i{k}_{jx}x},$$

1b $${\hat{H}}_{\xi }(x,y,z,\omega )=\sum _{j}\,{\hat{H}}_{j\xi }(z,\omega )\,{e}^{i{k}_{jx}x}\,(\xi =x,\,z),$$

Noise current density ${\hat{J}}_{y}({\boldsymbol{r}},\omega )$ and corresponding bosonic vector field ${\hat{f}}_{y}({\boldsymbol{r}},\omega )$ can be expressed in a similar way. The periodic relative permittivity can be also expanded as $\varepsilon (x,y,z,\omega )=\sum _{j}{\varepsilon }_{j}(\omega ){e}^{i{G}_{j}x}$. Here the wave vector is *k*_*jx*_ = *k*_*x*_ + *G*_*j*_, *G*_*j*_ = *jb*. The integer *j* in all the previous expressions is taken as *j* = 0, −1, 1, …, −*N*, *N*. In principle, the indices *j* should run from −∞ to +∞, but in numerical practice, truncation over a certain order is necessary. The number of the values of *j* is $M=2N+1$, ${\hat{E}}_{jy}(z,\omega )$ and ${\hat{H}}_{j\xi }(z,\omega )$ are expansion coefficients of the electric and magnetic fields, ${\varepsilon }_{j}(x,\omega )$, ${\hat{J}}_{jy}(z,\omega )$ and ${\hat{f}}_{jy}(z,\omega )$ represent expansion coefficients of the relative permittivity, noise current density and corresponding bosonic vector field. It is implicit in equations (1) that the Bloch theorem is satisfied for the one dimensional periodic medium.

The classical EM fields in the one dimensional periodic structures have already been studied in many previous works^29–31^. We borrow the ideas from these works to solve the quantum Maxwell equations, substitute the operator EM fields in quantum Maxwell equations for the plane waves expansion, thereby we find that the electric field ${\hat{E}}_{my}(z,\omega )$ obeys the partial differential equation

2 $$-\frac{{\partial }^{2}}{\partial {z}^{2}}{\hat{E}}_{my}(z,\omega )+\sum _{n}\,({k}_{mx}{k}_{nx}{\delta }_{m,n}-{\omega }^{2}/{c}^{2}{\varepsilon }_{m-n}){\hat{E}}_{ny}(z,\omega )=i\omega {\mu }_{0}{\hat{J}}_{my}(z,\omega ).$$

In order to solve the electric field in this equation, we rewrite it as the following matrix form

3 $$(-\frac{{\partial }^{2}}{\partial {z}^{2}}+P(\omega )){\hat{E}}_{y}(z,\omega )=i\omega {\mu }_{0}{\hat{J}}_{y}(z,\omega ),$$

where *P*(*ω*) is *M* × *M* matrix, ${\hat{E}}_{y}(z,\omega )$ and ${\hat{J}}_{y}(z,\omega )$ are both one column matrixes, ${\hat{E}}_{y}(z,\omega )=({\hat{E}}_{0y}(z,\omega ),{\hat{E}}_{-1y}(z,\omega ),$ ${\hat{E}}_{1y}(z,\omega ),\cdots {\hat{E}}_{Ny}(z,\omega ){)}^{T}$ and ${\hat{E}}_{y}(z,\omega )=({\hat{E}}_{0y}(z,\omega ),{\hat{E}}_{-1y}(z,\omega ),$ ${{\hat{E}}_{1y}(z,\omega ),\cdots {\hat{E}}_{Ny}(z,\omega ))}^{T}$, the superscript *T* means the transpose of the matrixes. We use the Green function method to solve the electric field ${\hat{E}}_{y}(z,\omega )$ in Eq. (3), the Green function^35^ *G*(*z*, *z’*, *ω*) is a *M* × *M* matrix, in order to solve it, the Fourier transforms of ${\hat{E}}_{y}(\kappa ,\omega )$ and ${\hat{J}}_{y}(\kappa ,\omega )$ of ${\hat{E}}_{y}(z,\omega )$ and ${\hat{J}}_{y}(z,\omega )$ should be introduced. Then we substitute these Fourier decomposition into Eq. (3) and obtain an equation in Fourier space shown as follows

4 $$({\kappa }^{2}I+P(\omega )){\hat{E}}_{y}(\kappa ,\omega )=i\omega {\mu }_{0}{\hat{J}}_{y}(\kappa ,\omega ).$$

The corresponding Green function *G*(*κ*, *κ*′, *ω*) for this equation satisfies

5 $$({\kappa }^{2}I+P(\omega ))G(\kappa ,\kappa ^{\prime} ,\omega )=I\delta (\kappa -\kappa ^{\prime} ),$$

here the Green function *G*(*κ*, *κ*′, *ω*) is also a *M* × *M* matrix which is the Fourier transform of *G*(*z*, *z*′, *ω*), *I* is the identity matrix, ${\hat{E}}_{y}(\kappa ,\omega )$ and ${\hat{J}}_{y}(\kappa ,\omega )$ are one column matrixes. The eigenvalues of the matrix *P*(*ω*) and the *M* × *M* matrix *S*(*ω*), whose *σ* th column (*S*_0*σ*_(*ω*), *S*_−1*σ*_(*ω*), *S*_1*σ*_(*ω*), …, *S*_−*Nσ*_(*ω*), *S*_*Nσ*_(*ω*))^*T*^ is the eigenvector corresponding to the eigenvalue −*κ*_*σ*_^2^(*ω*), can be obtained simultaneously, *κ*_*σ*_(*ω*) is the wavevector along z direction. The matrix *S*(*ω*) satisfies

6 $$\mathop{\sum }\limits_{\sigma =0}^{N}\,{S}_{m\sigma }(\omega ){S}_{n\sigma }^{\ast }(\omega )\delta (\kappa -\kappa ^{\prime} )={\delta }_{mn}\delta (\kappa -\kappa ^{\prime} ).$$

Then the Green function $G(\kappa ,\kappa ^{\prime} ,\omega )$ in Eq. (5) can be calculated based on Eq. (6)

7 $${G}_{mn}(\kappa ,\kappa ^{\prime} ,\omega )=\mathop{\sum }\limits_{\sigma =0}^{N}\,\frac{{S}_{m\sigma }{S}_{n\sigma }^{\ast }\delta (\kappa -\kappa ^{\prime} )}{(\kappa -{\kappa }_{\sigma }-i\delta )(\kappa +{\kappa }_{\sigma }+i\delta )}$$

where *δ* is a positive infinitesmal, the dependence on *ω* is implicit for *κ*_*σ*_(*ω*) and *S*(*ω*). The Green function *G*(*z*, *z’*, *ω*) can be calculated by integrating *G*(*κ*, *κ*′, *ω*) over *κ* and *κ*′

8 $${G}_{mn}(z,z^{\prime} ,\omega )=i\mathop{\sum }\limits_{\sigma =0}^{N}\,\frac{{S}_{m\sigma }{S}_{n\sigma }^{\ast }}{2{\kappa }_{\sigma }}{e}^{i{\kappa }_{\sigma }|z-z^{\prime} |},$$

the residue theorem is used in the calculation of this integral. Based on this Green function the field $\hat{E}$_*my*_(*z*, *ω*) can be solved

9 $${\hat{E}}_{my}(z,\omega )=i{\mu }_{0}\omega \sum _{\sigma }\,{S}_{m\sigma }[{e}^{i{\beta }_{\sigma }z}{\hat{o}}_{\sigma y}^{+}(z,\omega )+{e}^{-i{\beta }_{\sigma }z}{\hat{o}}_{\sigma y}^{-}(z,\omega )],$$

here the amplitude operators ${\hat{o}}_{\sigma y}^{+}(z,\omega )$ and ${\hat{o}}_{\sigma y}^{-}(z,\omega )$ are introduced, which propagate forward (along +z direction) and backward (along −z direction), respectively,

10a $${\hat{o}}_{\sigma y}^{+}(z,\omega )=i{\int }_{-\infty }^{z}\,dz^{\prime} \sum _{n}\,\frac{{S}_{n\sigma }^{\ast }}{2{k}_{\sigma }}{e}^{-i{\beta }_{\sigma }z}{e}^{i{\kappa }_{\sigma }(z-z^{\prime} )}{\hat{J}}_{ny}(z^{\prime} ,\omega ),$$

10b $${\hat{o}}_{\sigma y}^{-}(z,\omega )=i{\int }_{z}^{\infty }\,dz^{\prime} \sum _{n}\,\frac{{S}_{n\sigma }^{\ast }}{2{k}_{\sigma }}{e}^{i{\beta }_{\sigma }z}{e}^{-i{\kappa }_{\sigma }(z-z^{\prime} )}{\hat{J}}_{ny}(z^{\prime} ,\omega ),$$

where we assume the wave vector *κ*_*σ*_ = *β*_*σ*_ + *iγ*_*σ*_, *β*_*σ*_ and *γ*_*σ*_ are the real and imaginary parts of *κ*_*σ*_.

### Annihilation and creation operators

Based on the explicit expressions of amplitude operators, we have also studied the spatial evolution of the amplitude operators which is governed by quantum Langevin equations (see Supplementary Material), where the quantum noise associated with the damping is taken into account by operator Langevin noise sources. After consideration of the commutation relations of the operator noise current densities, we can get the commutation relations of the amplitude operators (see Supplementary Material), from the results we can see that the commutation relations of the amplitude operators with different orders may not be zero. A special case is considered *z* = *z’* and then we define a matrix *U*_*mn*_(*ω*) in this case

11 $$[{\hat{o}}_{my}^{\pm }(z,\omega ),{\hat{o}}_{ny}^{\pm \dagger }(z,\omega ^{\prime} )]={U}_{mn}(\omega ){e}^{\mp i{\beta }_{m}z\pm i{\beta }_{n}z}\delta (\omega -\omega ^{\prime} ).$$

The commutation relations of the photon annihilation operators with different orders should be equal to zero, so we introduce the photon annihilation operators ${\hat{a}}_{my}^{+}(z,\omega )$ and ${\hat{a}}_{my}^{-}(z,\omega )$, which are the linear superposition of the amplitude operators ${\hat{o}}_{my}^{+}(z,\omega )$ and ${\hat{o}}_{my}^{-}(z,\omega )$, respectively,

12 $${\hat{a}}_{my}^{\pm }(z,\omega ){e}^{\pm i{\beta }_{m}z}=\sum _{n}\,{[{X}^{\pm }(\omega )]}_{mn}^{-1}{\hat{o}}_{ny}^{\pm }(z,\omega ){e}^{\pm i{\beta }_{n}z}.$$

The matrixes of superposition coefficients *X*^+^ (*ω*) and *X*^−^(*ω*) are determined by the commutation relations of the bosonic photon annihilation and creation operators

13 $$[{\hat{a}}_{my}^{\pm }(z,\omega ),{\hat{a}}_{ny}^{\pm \dagger }(z,\omega ^{\prime} )]={\delta }_{mn}{e}^{-i({\beta }_{m}-{\beta }_{n})z}\delta (\omega -\omega ^{\prime} ),$$

Substituting in the left of the commutation relations in Eq. (13) for the photon operators the superposition forms (12), the coefficients *X*^+^ (*ω*) and *X*^−^(*ω*) can be determined by *U*(*ω*)

14 $${[{X}^{\pm }(\omega )]}^{-1}U(\omega ){[{X}^{\pm }(\omega )]}^{-1\dagger }=1.$$

It is clearly seen from Eq. (14) that *X*^+^ (*ω*) is equal to *X*^−^(*ω*) (*X*^+^ (*ω*) = *X*^−^(*ω*) = *X*(*ω*)).

So far, The EM field quantization in one-dimensional photonic crystal is fully completed, the final form of the electric field can be written as in matrix

15 $${\hat{E}}_{y}(z,\omega )=i{\mu }_{0}\omega SX[{e}^{i\beta z}{\hat{a}}_{y}^{+}(z,\omega )+{e}^{-i\beta z}{\hat{a}}_{y}^{-}(z,\omega )].$$

Here${e}^{\pm i\beta z}$ are the diagonal matrixes $diag({e}^{\pm i{\beta }_{0}z},\,{e}^{\pm i{\beta }_{-1}z},{e}^{\pm i{\beta }_{1}z}\cdots {e}^{\pm i{\beta }_{N}z})$ which describe the propagation of the quantum light, ${\hat{a}}_{y}^{\pm }(z,\omega )$ are one column matrixes ${({\hat{a}}_{0y}(z,\omega ),{\hat{a}}_{-1y}(z,\omega ),{\hat{a}}_{1y}(z,\omega ),\cdots {\hat{a}}_{Ny}(z,\omega ))}^{T}$. Here ${\hat{a}}_{0y}(z,\omega )$ is the radiation order and the others are high orders. The matrix *S* connects the amplitude operators in different orders with the electric field operators in different orders, it is not unity matrix in the grating, which reveal that there is interaction between different orders in this case. When the model degenerates to the homogeneous case, the matrix *S* is unity and *X* is diagonal, then our theory can degenerate successfully to the the corresponding results of the previous work^36–38^ of other authors who considered the EM quantization in the radiation order in the normal propagation case (see Supplementary Material).

### Quantum optical input-output relation for grating

**Figure 2:**
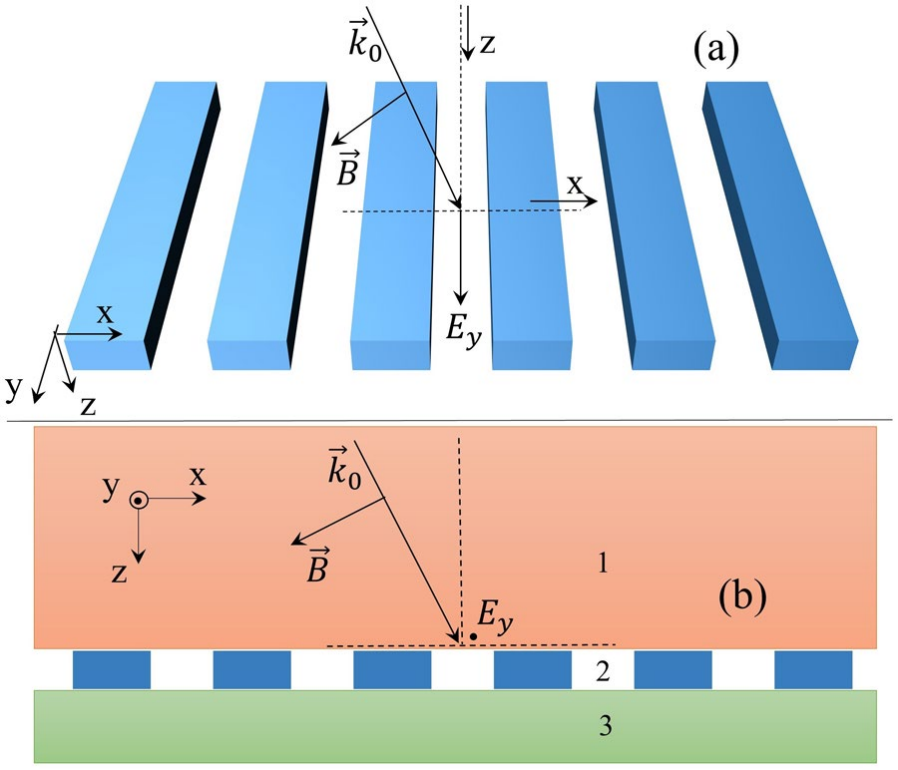
Scheme of the grating (marked by 2) with permittivity ${\varepsilon }^{2}(x,\omega )$ shown in (**a**) (oblique view) and (**b**) (side view) with thickness l embedded in homogeneous dielectrics (marked by 1 and 3) ${\varepsilon }^{1}(\omega )$ and ${\varepsilon }^{3}(\omega )$. The semi-infinite orange area up the grating is indicated by 1 and the semi-infinite green area down the grating is indicated by 3, the grating is located between these two semi-infinite areas.

Now we turn to the problem of propagation of the quantized field^39^ through 1D periodic dielectric slab—1D grating—embedded in two semi-infinite homogeneous dielectrics, which is shown in Fig. 2. The dielectric function is expressed as

16 $$\varepsilon (\overrightarrow{r},\omega )=\Theta (\,-\,z-l/2){\varepsilon }^{1}(\omega )+[\Theta (z+l/2)-\Theta (z-l/2)]{\varepsilon }^{2}(x,\omega )+\Theta (z-l/2){\varepsilon }^{3}(\omega ).$$

the superscripts 1, 2, 3 represent three corresponding regions.

The input-output relation of annihilation operators in transfer matrix form for the grating can be obtained by using EM boundary condition and solution of quantum Langevin equation (see Supplementary Material)

17 $$(\begin{array}{c}{\hat{a}}^{3+}(l/2,\omega )\\ {\hat{a}}^{3-}(l/2,\omega )\end{array})=(\begin{array}{cc}{T}_{11} & {T}_{12}\\ {T}_{21} & {T}_{22}\end{array})(\begin{array}{c}{\hat{a}}^{1+}(-l/2,\omega )\\ {\hat{a}}^{1-}(-l/2,\omega )\end{array})+(\begin{array}{cc}{t}_{11} & {t}_{12}\\ {t}_{21} & {t}_{22}\end{array})(\begin{array}{c}{\hat{G}}^{+}(\omega )\\ {\hat{G}}^{-}(\omega )\end{array}),$$

here the columns ${\hat{g}}^{+}(\omega )$ and ${\hat{g}}^{-}(\omega )$ are associated with the grating.

The commutation relations of ${\hat{G}}^{\pm }(\omega )$ with different orders may not be zero, in light of mentioned theoretical discussion we construct the new operators ${\hat{g}}_{n}^{+}(\omega )$ and ${\hat{g}}_{n}^{-}(\omega )$ associated with the slab which satisfy the bosonic commutation relations. Firstly, we define ${\hat{G}}_{n}^{0+}(\omega )$ and ${\hat{G}}_{n}^{0-}(\omega )$ to ensure that they commute with each other

18 $${\hat{G}}_{n}^{0\pm }(\omega )=-\,{\hat{G}}_{n}^{-}(\omega )\pm {\hat{G}}_{n}^{+}(\omega ),$$

After some calculation, the related commutation relations are listed in the following, moreover, the matrix *V* is introduced further

19a $$\begin{array}{rcl}[{\hat{G}}_{m}^{0\pm }(\omega ),{\hat{G}}_{n}^{0\pm \dagger }(\omega \text{'})] & = & \sqrt{\frac{1}{{\gamma }_{m}^{2}{\gamma }_{n}^{2}}}\sum _{p,q}\,\frac{{S}_{pm}^{2\ast }}{2{\kappa }_{m}^{2}}\frac{{S}_{qn}^{2}}{2{\kappa }_{n}^{2\ast }}{\int }_{-l/2}^{l/2}\,dz\text{'}[{e}^{i({\kappa }_{m}^{2}-{\kappa }_{n}^{2\ast })z^{\prime} }\pm {e}^{i({\kappa }_{m}^{2}+{\kappa }_{n}^{2\ast })z^{\prime} }]{\rho }_{p-q}^{2}(\omega )\\  &  & \,\times \delta (\omega -\omega ^{\prime} ={V}_{mn}(\omega )\delta (\omega -\omega ^{\prime} )\end{array},$$

19b $$[{\hat{G}}_{m}^{0+}(\omega ),{\hat{G}}_{n}^{0-\dagger }(\omega ^{\prime} )]=0\,{\rm{and}}\,[{\hat{G}}_{m}^{0-}(\omega ),{\hat{G}}_{n}^{0+\dagger }(\omega \text{'})]=0.$$

Secondly, we construct new operators ${\hat{g}}_{m}^{+}(\omega )$ and ${\hat{g}}_{n}^{-}(\omega )$ as linear superposition of operators ${\hat{G}}_{n}^{0+}(\omega )$ and ${\hat{G}}_{n}^{0-}(\omega )$ in the similar way to construct the photon operators

20 $${\hat{g}}_{m}^{\pm }(\omega )=\sum _{n}\,{Y}_{mn}^{-1\pm }(\omega ){\hat{G}}_{j}^{0\pm }(\omega ),$$

The new operators should fulfill the bosonic commutation relations

21 $$[{\hat{g}}_{m}^{\pm }(\omega ),{\hat{g}}_{n}^{\pm \dagger }(\omega ^{\prime} )]={\delta }_{mn}\delta (\omega -\omega ^{\prime} ),$$

Similarly, the coefficients of *Y*^+^ (*ω*) and *Y*^−^ (*ω*) can be determined from the above equations by substituting the Eqs. (20) into (21)

22 $${[{Y}^{\pm }(\omega )]}^{-1}{V}^{\pm }(\omega ){[{Y}^{\pm }(\omega )]}^{-1\dagger }=1.$$

Finally, the quantum optical input-output relation expressed in the transfer matrix form can be transformed to the scattering matrix *Q*_*mn*_(*mn* = 11, 12, 21, 22)

23 $$(\begin{array}{c}{\hat{a}}^{3+}(\frac{1}{2}l,\omega )\\ {\hat{a}}^{1-}(-\frac{1}{2}l,\omega )\end{array})=(\begin{array}{cc}{Q}_{11} & {Q}_{12}\\ {Q}_{21} & {Q}_{22}\end{array})(\begin{array}{c}{\hat{a}}^{1+}(-\frac{1}{2}l,\omega )\\ {\hat{a}}^{3-}(\frac{1}{2}l,\omega )\end{array})+(\begin{array}{cc}{A}_{11} & {A}_{12}\\ {A}_{21} & {A}_{22}\end{array})(\begin{array}{c}{\hat{g}}^{+}(\omega )\\ {\hat{g}}^{-}(\omega )\end{array}).$$

So far we construct the relation between the output annihilation operators ${\hat{a}}^{3+}(l/2,\omega )$, ${\hat{a}}^{1-}(-l/2,\omega )$ and the input annihilation operators ${\hat{a}}^{1+}(-l/2,\omega )$, ${\hat{a}}^{3-}(l/2,\omega )$ and the bosonic excitations associated with the slab ${\hat{g}}^{+}(\omega )$, ${\hat{g}}^{-}(\omega )$. The new operators ${\hat{g}}^{+}(\omega )$ and ${\hat{g}}^{-}(\omega )$ play the role of the noise sorces associated with the damping in the input-output relation. When we consider the special case of homogeneous dielectric, the input-output relation and related commutation relations can be also derived back to the previous study^37,38^.

Then we can derive the commutation relations between the output photon operators based on the input-output relation together with the known commutation relations between the input photon operators. After deliberate and straightforward calculation the results can be written in matrix form

24 $$(\begin{array}{cc}{c}_{11} & {c}_{12}\\ {c}_{21} & {c}_{22}\end{array})=(\begin{array}{cc}{Q}_{11} & {Q}_{12}\\ {Q}_{21} & {Q}_{22}\end{array}){(\begin{array}{cc}{Q}_{11} & {Q}_{12}\\ {Q}_{21} & {Q}_{22}\end{array})}^{\dagger }+(\begin{array}{cc}{A}_{11} & {A}_{12}\\ {A}_{21} & {A}_{22}\end{array}){(\begin{array}{cc}{A}_{11} & {A}_{12}\\ {A}_{21} & {A}_{22}\end{array})}^{\dagger }.$$

Here the matrixes shown in the above equation are defined as ${c}_{11,mn}=[{\hat{a}}_{m}^{3+}(l/2,\omega ),{\hat{a}}_{n}^{3+\dagger }(l/2,\omega )],{c}_{12,mn}=$ $[{\hat{a}}_{m}^{3+}(l/2,\omega ),{\hat{a}}_{n}^{1-\dagger }(-l/2,\omega )]$, ${c}_{21,mn}=[{\hat{a}}_{m}^{1-}(-l/2,\omega ),{\hat{a}}_{n}^{3+\dagger }(l/2,\omega )]$ and ${c}_{22,mn}=[{\hat{a}}_{m}^{1-}(-l/2,\omega ),$ ${\hat{a}}_{n}^{1-\dagger }(-l/2,\omega )]$. In the following we give the results about the values of commutation relations for the uniform slab and grating immersed in air. The grating refers to alternating dielectric bar and air, the period and bar width of the grating are denoted by Λ and *w*, the relative permittivity of the dielectric bar is marked by *ε*, the thicknesses of the uniform slab and grating are both denoted by *l*, the wavelength and incident angle of the EM field are represented by *λ* and *θ*. Because of the symmetry of this model, *c*_11,*mn*_ = *c*_22,*mn*_, *c*_12,*mn*_ = *c*_21,*mn*_.

The diagonal elements of matrixes *c*_11_, *c*_12_, *c*_21_ and *c*_22_ are real number which can be seen from Eq. (24). For the uniform dielectric the matrix *X* is diagonal, if the uniform dielectric is lossless, the coefficients *X*_*mn*_ approach zero for higher orders (*m* ≠ 0), so only the radiated (*m* = 0) annihilation and creation operators are physically significant. Hence, we are interested in *c*_11,00_, *c*_12,00_, *c*_21,00_ and *c*_22,00_ in radiation order for our model.

**Figure 3:**
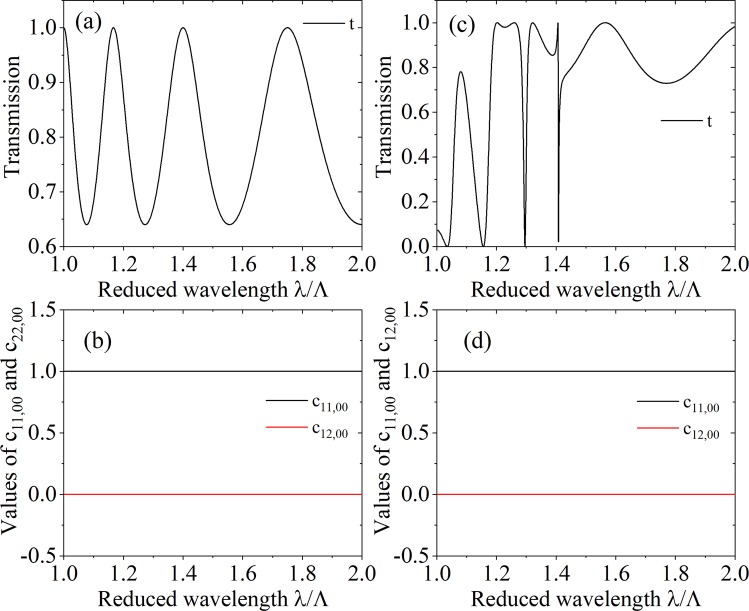
The transmission and values of commutation relations, ***c***_11,00_ and ***c***_12,00_, of the medium layer without loss as a function of the reduced wavelength *λ*/*Λ*. The transmission is studied in (**a**) and (**c**), ***c***_11,00_ and ***c***_12,00_ are studied in (**b**) and (**d**), the uniform slab with relative permittivity 4.0 is considered for (**a**) and (**b**), and the grating with filling factor *w*/Λ = 0.6 is considered for (**c**) and (**d**), ${\boldsymbol{\varepsilon }}=4.0$, *θ* = 0°. The thicknesses of the uniform slab and grating are both equal to *d* = 1.75*Λ*.

**Figure 4:**
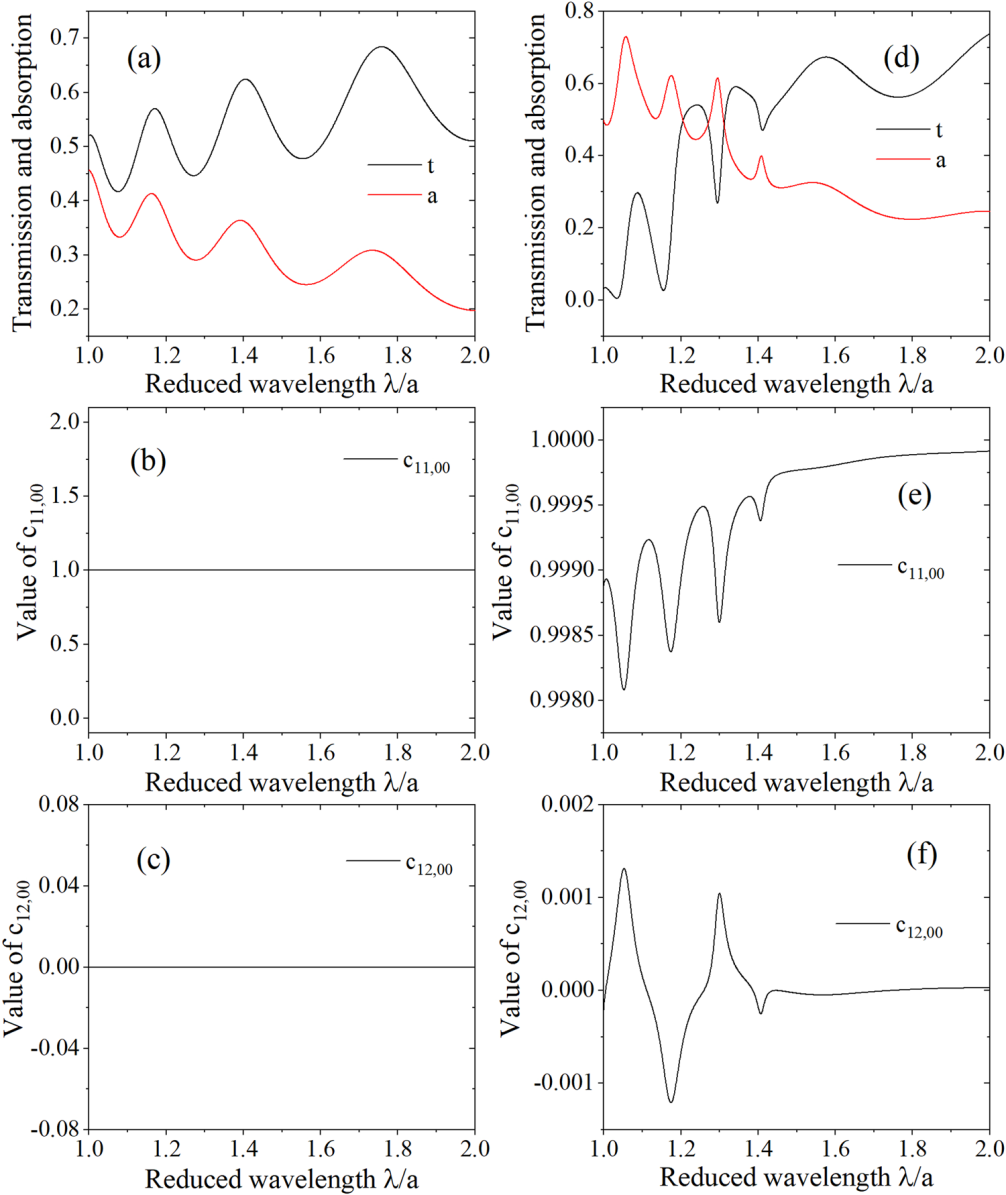
The transmission and values of commutation relations, *c*_11,00_ and *c*_12,00_, of the medium layer with loss as a function of the reduced wavelength *λ*/Λ. The transmission is studied in (**a**) and (**d**), *c*_11,00_ is studied in (**b**) and (**e**), and *c*_12,00_ is studied in (**c**) and (**f**), the uniform slab with relative permittivity (4.0, 0.1) is considered for (**a**), (**b**) and (**c**), and the grating with filling factor *w*/Λ = 0.6 is considered for (**d**–**f**), ${\boldsymbol{\varepsilon }}=(4.0,0.1)$, *θ* = 0°. The thicknesses of the uniform slab and grating are both equal to *d* = 1.75*Λ*.

In Fig. 3 we consider the transmission and values of commutation relations, *c*_11,00_ and *c*_22,00_, for the lossless uniform slab and lossless grating as a function of the reduced wavelength *λ*/Λ. The corresponding case of lossy layer is shown in Fig. 4. For uniform slab, no matter it is lossless or lossy, *c*_11,00_ = 1 and *c*_12,00_ = 0 hold, which can be seen from Figs. 3(b), 4(b,c), that means the output photons satisfy bosonic commutation relation and the annihilation operators for different channels commute with each other, these results coincide with the former work. For grating, only when it is lossless, *c*_11,00_ = 1 and *c*_12,00_ = 0, which can be seen from Fig. 3(d), when it is lossy, these equations are not true in this case, that is to say, *c*_11,00_ ≠ 1 and *c*_12,00_ ≠ 0, which can be seen from 4(e,f). After comparing the four different models, we find that the physical origin of this inequality is that the excitations, ${\hat{g}}_{m}^{+}(\omega )$ and ${\hat{g}}_{m}^{-}(\omega )$, in different orders interact with each other. Not only that, from Fig. 4(d–f) we also find that near the guided resonance, which is the Fano resonance in our optical model, obvious resonance and deviation of *c*_11,00_ and *c*_12,00_, the deviation means that the departure of *c*_11,00_ value from 1 and *c*_12,00_ value from 0. It can be also clearly seen that at the reduced wavelength *λ*/*a* > 1.5, there is no guided resonance, while there is also no resonance for *c*_11,00_ and *c*_12,00_ and the deviation decreases.

The Heisenberg picture is implied in the quantization theory, when the theory is converted to Schördinger picture, we can understand the phenomenon further, which is the deviation of the bosonic commutation relations for the output photon operators in the lossy grating. In the Schördinger picture, the evolution operator is no longer unitary with respect to the radiated order which can be derived from the input-output relation^40,41^, so the transformation of the quantum states is non-unitary. From Fig. 4(e,f) we can see that the phenomenon of deviation is very small (~10^−3^).

**Figure 5:**
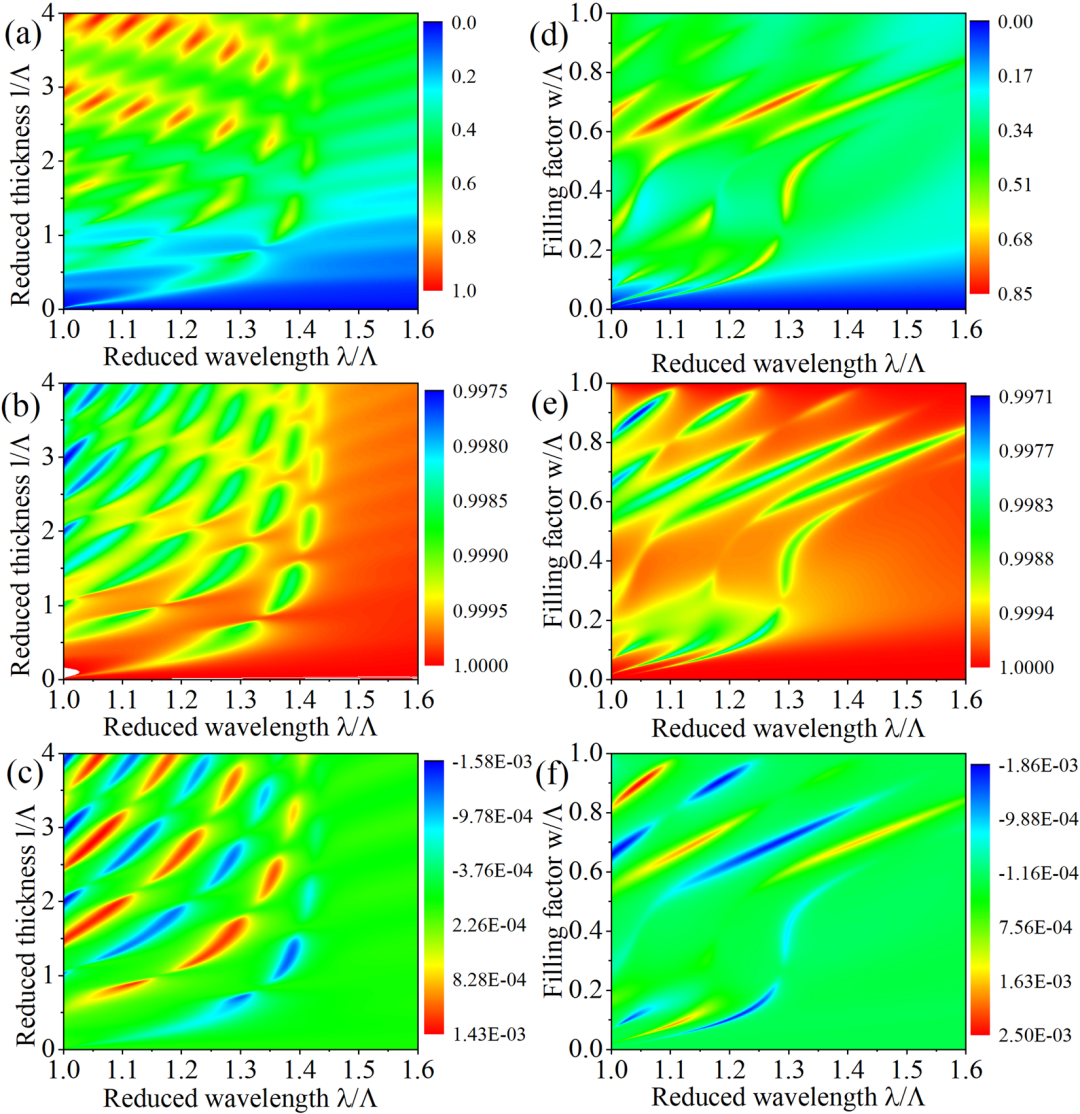
The absorption, *c*_11,00_ and *c*_12,00_ for the grating when the geometric parameters are changed: (**a**–**c**) show the absorption, *c*_11,00_, and *c*_12,00_ as functions of the reduced wavelength *λ*/Λ and the reduced thickness *l*/Λ, the width of the grating is chosen as *w* = 0.6*Λ*. (**d**–**f**) depict the absorption, *c*_11,00_ and *c*_12,00_ as functions of the reduced wavelength *λ*/Λ and the filling factor *w*/Λ, the thickness of the grating is chosen as *l* = 1.75*Λ*. For all these cases, The relative permittivity of the grating is ${\boldsymbol{\varepsilon }}=(4,\,0.1)$ and the incident photons propagate normally *θ* = 0°.

**Figure 6:**
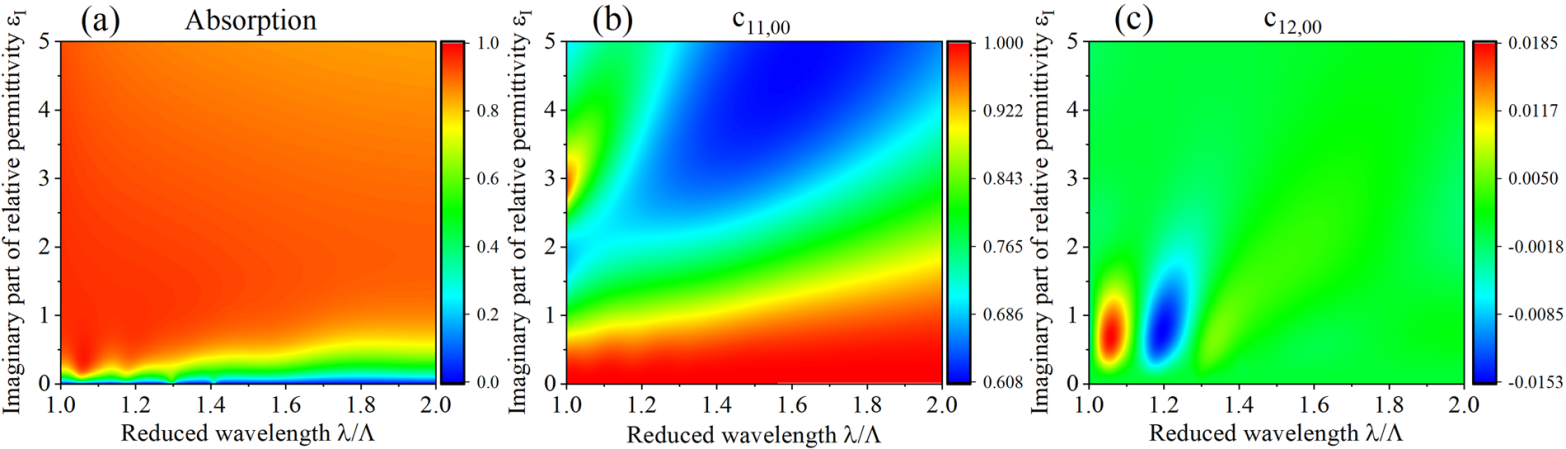
The absorption (**a**), *c*_11,00_ (**b**) and *c*_12,00_ (**c**) for the grating as functions of reduced wavelength *λ*/Λ and imaginary part of the relative permittivity ${{\boldsymbol{\varepsilon }}}_{{\boldsymbol{I}}}$. The real part of the relative permittivity is fixed at 4.0, the thickness and width of the grating are chosen as *l* = 1.75*Λ*, and *w* = 0.6*Λ*, respectively, the incident photons propagate normally *θ* = 0°.

Now we tune *c*_11,00_ and *c*_12,00_, which describe the transformation of the photon states, by change the parameters in our model. Compared to the classical optics the Fano resonance can appear in the grating for quantum light which can be seen from Fig. 5(a,d), near the resonant absorption is notable. The peak and valley of *c*_11,00_ and *c*_12,00_ are coincident with the resonant absorption, the sign of *c*_12,00_ is opposite to that near the adjacent resonant curve. We can see that the deviations of *c*_11,00_ from 1 and *c*_12,00_ from 0 are closely related to the Fano resonance in the grating. It is clearly seen in Fig. 5 that these deviations are very weak (~10^−3^) due to the weak absorption (the imaginary part of the relative permittivity is only ${\varepsilon }_{I}=0.1$). In Fig. 6 it is implied that the deviation of *c*_11,00_ from 1 rises by increasing the imaginary part of the relative permittivity which is closely related to the effect of absorption, this devation can reach about 0.4. When *ε*_*I*_ is located at (0.3, 1.5) the deviation of *c*_12,00_ from 0 can be increased about 1 order of magnitude (~10^−2^). *ε*_*I*_ variation leads to notable effect of ${\hat{g}}_{m}^{+}(\omega )$ and ${\hat{g}}_{m}^{-}(\omega )$ in higher orders on the output photon operators in radiation order, so we can enhance the effect of the nonunitary evolution obviously by changing the imaginary part of the relative permittivity of the grating.

We have also calculated the transmission, reflection and absorption for photon number density, which are equal to that in classical optics. The sharp resonance, guided resonance, which appears in the grating for classical light can also emerge for quantum light. At the resonance, 100% relative numbers of the outgoing photons in output channels are exhibited and near 100% absorption is realized for the lossy grating, the Q factor is high and the lifetime is long. From the asymptotic behavior of the resonance, some embedded resonances with zero linewidth can be found, these embedded resonances possess infinite high Q factor and infinite long lifetime, and are called light bound states in the continuum (BICs) which have attracted much attention in recent years in classical optics^13–15^. In our work, we also find the light BICs in quantum optics in theory. In classical optics, many applications of the grating are developed because of their excellent optical properties^42,43^, we believe that the grating can be applied in various areas of quantum optics, such as propagation of non-classical light, quantum state transformation, spontaneous emission of a nearby scatter and so on, these will be our next tasks.

## Conclusion

We give the Green function and the EM field quantization for 1D periodic, dispersive and absorbing dielectric bulk medium firstly. The EM field are expanded in plane waves and are inserted to the quantum Maxwell equations, the Green function is solved, furthermore the electric field is quantized and the amplitude annihilators are established. The commutation relations of these amplitude operators in our periodic bulk system are calculated out based on the previous known commutation relations of the operator noise current density, we find that the amplitude operator don’t commute with the its Hermite operator with different order, which is quite different from the homogeneous dielectric case. Then we construct the photon annihilation operators by linear superposition of the amplitude operators. The quantum Langevin equations which determine the spatial evolution of the amplitude operators in our bulk system are provided and studied.

The quantum input-output relation for the grating is also derived, the output field operators can be described in terms of input field operators and noise sources associated with the loss in the gratings. We find that the conventional commutation relations are satisfied, ${c}_{11,00}=[{\hat{a}}_{0}^{3+}(l/2,\omega ),{\hat{a}}_{0}^{3+\dagger }(l/2,\omega )]=1$ and ${c}_{12,00}=[{\hat{a}}_{0}^{3+}(l/2,\omega ),$, ${\hat{a}}_{0}^{1-\dagger }(-l/2,\omega )]=0$for uniform slab or lossless grating, but for lossy grating, these relations do not hold, these phenomena originate from the interaction between the output photon in radiation order and the excitations in higher orders. The excellent quantum optical properties of the grating are also found and discussed. We believe our work is very beneficial for the control and regulation of the quantum light based on gratings.

## Supplementary information

Supplementary information
