# Supplementary material for: Electromagnetic field quantization and quantum optical input-output relation for grating
Source: Sci Rep. 2019 Dec 27;9:19992. doi: 10.1038/s41598-019-56197-1 (PMC6934606; doi:10.1038/s41598-019-56197-1)
Supplement: Supplementary file 1 — Supplementary information [file 41598_2019_56197_MOESM1_ESM.pdf]

Supplementary Material for Electromagnetic field quantization and quantum optical input-output relation for grating

Tiecheng Wang[1*]

[1]*College of Physics and Electronic Engineering, Shanxi University, 030006, Taiyuan, China*

** E-Mail address: tcwang@sxu.edu.cn*

Quantum Langevin equations The amplitude operators $\hat{o}_{\sigma y}^{+}(z,\omega)$ and $\hat{o}_{\sigma y}^{-}(z,\omega)$ defined in the main text maybe regarded as system operators that describe the space-dependent amplitudes of the monochromatic EM field in 1D periodic, dispersive and lossy dielectric medium. These amplitudes are exponentially damped out in space and the characteristic length is given by γ_{σ}^{-1}. The spatial evolution of the amplitude operators is governed by quantum Langevin equations, where the quantum noise associated with the damping is taken into account by operator Langevin noise sources, the spatial evolutions of the amplitude operators can be derived from their definition

$$\frac{\partial \hat{o}_{\sigma y}^{\pm}(z,\omega)}{\partial z} = \mp\gamma_{\sigma}\hat{o}_{\sigma y}^{\pm}(z,\omega) + \hat{L}_{\sigma}^{\pm}(z,\omega), \tag{S1}$$

these are quantum Langevin equations where the operator Langevin noise sources are given by

$$\hat{L}_{\sigma}^{\pm}(z,\omega) = \pm i\sum_{n}\frac{S_{n\sigma}^{*}}{2\kappa_{\sigma}}e^{\mp i\beta_{\sigma}z}\hat{J}_{ny}(\mathrm{z},\omega), \tag{S2}$$

The commutators $\left[\hat{L}_{m}^{+}(z,\omega),\hat{L}_{n}^{+\dagger}(z',\omega')\right]$and $\left[\hat{L}_{m}^{-}(z,\omega),\hat{L}_{n}^{-\dagger}(z',\omega')\right]$ can be calculated based on Eqs. (S2)

$$\left[\hat{L}_{m}^{\pm}(z,\omega),\hat{L}_{n}^{\pm\dagger}(z',\omega')\right] = e^{\mp i\beta_{m}z\pm i\beta_{n}z'}\sum_{\alpha\beta}\frac{S_{\alpha m}^{*}}{2\kappa_{m}}\frac{S_{\beta n}}{2\kappa_{n}^{*}}\rho_{\alpha-\beta}(\omega)\delta(z-z')\delta(\omega-\omega'), \tag{S3}$$

The commutation relations between the operators $\hat{L}_{m}^{+}(z,\omega)$, $\hat{L}_{m}^{-}(z,\omega)$ and the amplitude operators are given by

$$\left[\hat{o}_{m}^{\pm}(z,\omega),\hat{L}_{n}^{\pm\dagger}(z',\omega')\right] = 0 \quad \text{if} \quad z-z' \lesseqgtr 0, \tag{S4}$$

Integrate the amplitude operators in equation (S4) over *z*, we get the solutions

$$\hat{o}_{\sigma y}^{\pm}(z,\omega) = e^{\mp\gamma_{\sigma}(z-z')}\hat{o}_{\sigma y}^{\pm}(z',\omega) + \int_{z'}^{z}d\tilde{z}e^{-\gamma_{\sigma}(z-\tilde{z})}\hat{L}_{\sigma}^{+}(\tilde{z},\omega) \ \text{if} \quad z-z' \gtreqless 0, \tag{S5}$$

In order to compare the quantum Langevin equations with the previous works of other authors. We also consider the homogeneous dielectric and only the radiation order in the normal propagation case, following our derivation, the spatial evolutions of the photon operators $\hat{a}_{y}^{+}(z,\omega)$ and $\hat{a}_{y}^{-}(z,\omega)$ can be expressed as

$$\frac{\partial \hat{a}_{y}^{\pm}(z,\omega)}{\partial z} = \mp\gamma\hat{a}_{y}^{\pm}(z,\omega) \pm i\sqrt{\frac{\omega}{c}2n_{i}(\omega)}e^{\mp i\omega n_{r}(\omega)z/c}\hat{f}_{y}(z), \tag{S6}$$

These quantum Langevin equations coincide with that in the predecessors' work, the other results in this part can be also derived back to the related studies in the previous work successfully.

Commutation relations of amplitude operators The commutation relations of the operator noise current density need to be calculated in order to obtain the commutation relations of the amplitude operators, based on the relation between the source term $\hat{\boldsymbol{J}}(\vec{r},\omega)$ and the bosonic vector field $\hat{\boldsymbol{f}}(\boldsymbol{r},\omega)$ and the commutation relations of the components of $\hat{\boldsymbol{f}}(\boldsymbol{r},\omega)$ we obtain

$$[\hat{J}_y(\boldsymbol{r},\omega),\hat{J}_y^{\dagger}(\boldsymbol{r}',\omega')] = \rho(\vec{r},\omega)\delta(\boldsymbol{r}-\boldsymbol{r}')\delta(\omega-\omega'), \quad \text{(S7a)}$$

$$[\hat{J}_y(\boldsymbol{r},\omega),\hat{J}_y(\boldsymbol{r}',\omega')] = [\hat{J}_y^{\dagger}(\boldsymbol{r},\omega),\hat{J}_y^{\dagger}(\boldsymbol{r}',\omega')] = 0, \quad \text{(S7b)}$$

here $\rho(\boldsymbol{r},\omega) = \frac{\varepsilon_0\hbar\omega^2}{\pi}\varepsilon_I(\boldsymbol{r},\omega)$, substituting in equations (S7) for the operator noise current density the in plane wave expression of the operator current density $\hat{J}_y(\boldsymbol{r},\omega)$, after a little lengthy calculation we get

$$[\hat{J}_{my}(z,\omega),\hat{J}_{ny}^{\dagger}(z',\omega')] = \rho_{m-n}(\omega)\delta(z-z')\delta(\omega-\omega'), \quad \text{(S8a)}$$

$$[\hat{J}_{my}(z,\omega),\hat{J}_{ny}(z',\omega')] = [\hat{J}_{my}^{\dagger}(z,\omega),\hat{J}_{ny}^{\dagger}(z',\omega')] = 0, \quad \text{(S8b)}$$

$$\hat{J}_{my}(z,\omega) = \sqrt{\frac{\varepsilon_0\hbar\omega^2}{\pi}}\sum_n \sqrt{\varepsilon_I}_n \hat{f}_{m-ny}(z,\omega), \quad \text{(S8c)}$$

where $\rho_{m-n}(\omega) = \frac{\varepsilon_0\hbar\omega^2}{\pi}\varepsilon_{Im-n}(\omega)$.

After some calculation, the commutation relations of the amplitude operators can be obtained based on the amplitude operator expressions and the commutation relations (S8)

$$[\hat{o}_{my}^{+}(z,\omega),\hat{o}_{ny}^{+\dagger}(z',\omega')] = \frac{i}{\kappa_m-\kappa_n^*}\sum_{\alpha\beta}\frac{S_{\alpha m}^*}{2\kappa_m}\frac{S_{\beta n}}{2\kappa_n^*}\rho_{\alpha-\beta}(\omega)\big[e^{i\beta_n z'-\gamma_m z-i\kappa_m z'}\theta(z-z')$$
$$+e^{-i\beta_m z+i\kappa_n^* z-\gamma_n z'}\theta(z'-z)\big]\delta(\omega-\omega'), \quad \text{(S9a)}$$

$$[\hat{o}_{my}^{-}(z,\omega),\hat{o}_{ny}^{-\dagger}(z',\omega')] = \frac{i}{\kappa_m-\kappa_n^*}\sum_{\alpha\beta}\frac{S_{\alpha m}^*}{2\kappa_m}\frac{S_{\beta n}}{2\kappa_n^*}\rho_{\alpha-\beta}(\omega)\,\big[e^{i\beta_m z-i\kappa_n^* z+\gamma_n z'}\theta(z-z')$$
$$+e^{-i\beta_n z'+\gamma_m z+i\kappa_m z'}\theta(z'-z)\big]\times\delta(\omega-\omega'), \quad \text{(S9b)}$$

$$[\hat{o}_{my}^{+}(z,\omega),\hat{o}_{ny}^{-\dagger}(z',\omega')] = \frac{i}{\kappa_m+\kappa_n^*}\sum_{\alpha\beta}\frac{S_{\alpha m}^*}{2\kappa_m}\frac{S_{\beta n}}{2\kappa_n^*}\left(e^{-i\kappa_n^* z+\gamma_n z'-i\beta_m z} - e^{-\gamma_m z-i\kappa_m z'-i\beta_n z'}\right)$$
$$\times\rho_{\alpha-\beta}(\omega)\delta(\omega-\omega')\theta(z-z'), \quad \text{(S9c)}$$

$$[\hat{o}_{my}^{-}(z,\omega),\hat{o}_{ny}^{+\dagger}(z',\omega')] = \frac{i}{\kappa_m+\kappa_n^*}\sum_{\alpha\beta}\frac{S_{\alpha m}^*}{2\kappa_m}\frac{S_{\beta n}}{2\kappa_n^*}\left(e^{i\kappa_n^* z-\gamma_n z'+i\beta_m z} - e^{\gamma_m z+i\kappa_m z'+i\beta_n z'}\right)$$
$$\times\rho_{\alpha-\beta}(\omega)\delta(\omega-\omega')\theta(z'-z), \quad \text{(S9d)}$$

The commutation relations between the amplitude operators or between the Hermitian conjugates of the amplitude operators are equal to zero due to (S8b). Here $\theta(z-z')$ is step function, which is equal to 1 if $z \geq z'$ or equal to zero if $z < z'$.

Electromagnetic field quantization in homogeneous dielectric When we consider the homogeneous dielectric which is a special case of periodic structure, the previous quantization theory can be applied, in this case, the matrix S is identity matrix and U is a diagonal matrix

$$U_{mn} = \frac{1}{2\gamma_m}\frac{1}{2\kappa_m}\frac{1}{2\kappa_m^*}\frac{\varepsilon_0\hbar\omega^2}{\pi}\varepsilon_I(\omega)\delta_{mn}, \quad \text{(S10)}$$

So the coefficient matrix X in homogeneous dielectric case can be expressed as

$$X_{mn} = \sqrt{\frac{\varepsilon_0\hbar\omega^2\varepsilon_I(\omega)}{8\pi\gamma_m\kappa_m\kappa_m}}\delta_{mn}, \quad \text{(S11)}$$

it can be seen that X is also a diagonal matrix. In homogeneous case, the amplitude operators $\hat{o}_{my}^{+}(z,\omega)$ and $\hat{o}_{my}^{-}(z,\omega)$ are equal to the annihilation operators $\hat{a}_{my}^{+}(z,\omega)$ and $\hat{a}_{my}^{-}(z,\omega)$ multiplied by a constant X_{mm}^{+}.

In Fig. S1 we plot the five coefficients X_{11}, X_{22}, X_{33}, X_{44} and X_{55} in air with small absorption, the real parts and imaginary parts of the coefficients as a function of the imaginary part of the relative permittivity are plotted in Fig. S1(a) and S1(b), respectively. It can be seen clearly

that the first coefficient X_{11} becomes a definite real number and higher order coefficients become zero as the imaginary part of the relative permittivity approaches zero. These results are in agreement with the former work[1].

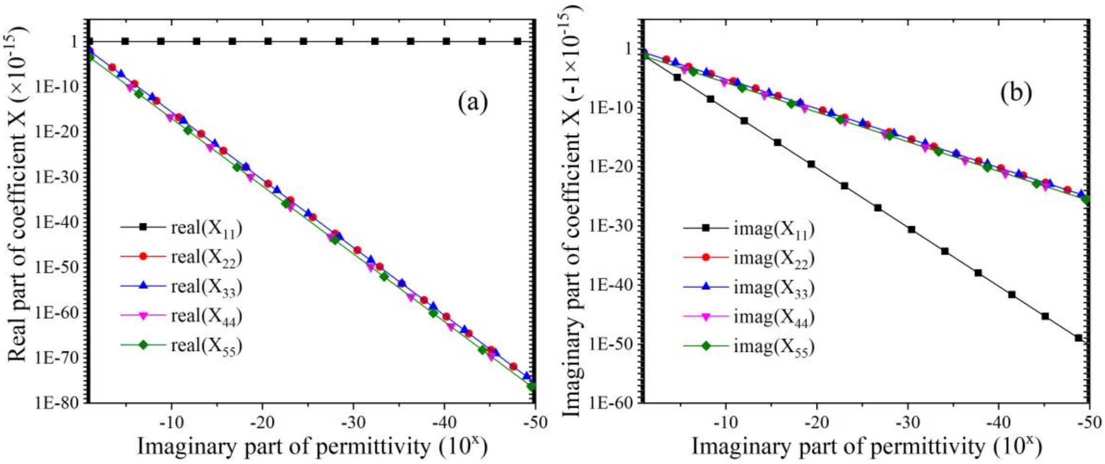

Figure S1. The real parts and imaginary parts of the coefficients X_{ii} $(i = 1, 2, 3, 4, 5)$ as a function of the imaginary part of the relative permittivity are plotted in (a) and (b), respectively. These results are multiplied by a same constant number. The real part of the permittivity is 1 and the reduced wavelength is 1.5.

In order to compare our theory with previous works which provide the EM quantization in radiattion mode for homogenous dielectric, following our derivation, the operator electric field in the radiation order $(m = 0)$ in the normal propagation case can be expressed as

$$\hat{E}_y(z,\omega) = i\sqrt{\frac{\hbar\omega}{4\pi\varepsilon_0 c n(\omega) n(\omega)}}\, n_r(\omega)\left[e^{i\omega n_r(\omega)z/c}\hat{a}_y^+(z,\omega) + e^{-i\omega n_r(\omega)z/c}\hat{a}_y^-(z,\omega)\right], \qquad (S12a)$$

$$\hat{a}_y^+(z,\omega) = i\sqrt{2\frac{\omega}{c}n_i(\omega)}\int_{-\infty}^{z} dz' e^{-i\omega n_r(\omega)z/c} e^{i\omega n(\omega)(z-z')/c}\hat{f}_y(z',\omega), \qquad (S12b)$$

$$\hat{a}_y^-(z,\omega) = i\sqrt{2\frac{\omega}{c}n_i(\omega)}\int_{z}^{\infty} dz' e^{i\omega n_r(\omega)z/c} e^{-i\omega n(\omega)(z-z')/c}\hat{f}_y(z',\omega), \qquad (S12c)$$

in the above equations we omit the subscript of the integer 0 because we only consider the radiation order. The commutation relations of annihilation and creation operators are derived back to the previous conclusions. These results are in agreement with the corresponding results of the previous work of other authors[2, 3.]

Quantum optical input-output relation for grating In the following we study the input-output relations for a grating of thickness l. Based on the quantized scheme the electric field operator in the *j*th $(j = 1, 2, 3)$ region can be written as

$$\hat{E}_{my}^j(z,\omega) = i\mu_0\omega\sum_n S_{mn}^j\left[e^{i\beta_n^j z}\hat{o}_n^{j+}(z,\omega) + e^{-i\beta_n^j z}\hat{o}_n^{j-}(z,\omega)\right]. \qquad (S13)$$

The corresponding magnetic field operator in the *j*th region is obtained from the quantum Maxwell equation

$$\hat{H}_{mx}(z,\omega) = -\sum_n S_{mn}^j\left[i\kappa_n^j e^{i\beta_n^j z}\hat{o}_n^{j+}(z,\omega) - i\kappa_n^j e^{-i\beta_n^j z}\hat{o}_n^{j-}(z,\omega)\right]. \qquad (S14)$$

The amplitude operators of the incoming fields from the bottom $(-\infty \le z \le -l/2)$ and top $(l/2 \le z \le \infty)$, $\hat{o}_n^{1+}(-l/2,\omega)$ and $\hat{o}_{nL}^{3-}(l/2,\omega)$ shown in Fig.3 can be written as

$$\hat{o}_n^{1+}(-l/2,\omega) = i\int_{-\infty}^{-l/2} d\tilde{z}\frac{1}{2k_n^1}e^{i\beta_n^1 l/2}e^{i\kappa_n^1(-l/2-\tilde{z})}\hat{J}_{ny}^1(\tilde{z},\omega), \tag{S15a}$$

$$\hat{o}_n^{3-}(l/2,\omega) = i\int_{l/2}^{\infty} d\tilde{z}\frac{1}{2k_n^3}e^{i\beta_n^3 l/2}e^{-i\kappa_n^3(l/2-\tilde{z})}\hat{J}_{ny}^3(\tilde{z},\omega). \tag{S15b}$$

The field operators in 1th, 2th and 3th regions are, of course, not independent but are related by the boundary conditions at the two interfaces. At the interface $z = l/2$, we can express the boundary condition from the equations (S13) and (S14) and write it in matrix form

$$\begin{pmatrix} S^3 & S^3 \\ T^3 & -T^3 \end{pmatrix}\begin{pmatrix} e^{i\beta^3 l/2}X^3\hat{a}^{3+}(l/2,\omega) \\ e^{-i\beta^3 l/2}X^3\hat{a}^{3-}(l/2,\omega) \end{pmatrix} = \begin{pmatrix} S^2 & S^2 \\ T^2 & -T^2 \end{pmatrix}\begin{pmatrix} e^{i\beta^2 l/2}\hat{o}^{2+}(l/2,\omega) \\ e^{-i\beta^2 l/2}\hat{o}^{2-}(l/2,\omega) \end{pmatrix}. \tag{S16}$$

A similar relation holds at the interface $z = -l/2$

$$\begin{pmatrix} S^2 & S^2 \\ T^2 & -T^2 \end{pmatrix}\begin{pmatrix} e^{-i\beta^2 l/2}\hat{o}^{2+}(-l/2,\omega) \\ e^{i\beta^2 l/2}\hat{o}^{2-}(-l/2,\omega) \end{pmatrix} = \begin{pmatrix} S^1 & S^1 \\ T^1 & -T^1 \end{pmatrix}\begin{pmatrix} e^{-i\beta^1 l/2}X^1\hat{a}^{1+}(-l/2,\omega) \\ e^{i\beta^1 l/2}X^1\hat{a}^{1-}(-l/2,\omega) \end{pmatrix}, \tag{S17}$$

here $e^{\pm i\beta^j z}o^{j\pm}(z,\omega) = \left(e^{\pm i\beta_0^j z}\hat{o}_0^{j\pm}(z,\omega), e^{\pm i\beta_{-1}^j z}\hat{o}_{-1}^{j\pm}(z,\omega)\cdots e^{\pm i\beta_N^j z}\hat{o}_N^{j\pm}(z,\omega)\right)^{\mathrm{T}}$, the elements of the matrix T^j $(j = 1,2,3)$ are expressed as $T_{mn}^j = \frac{i}{\omega\mu_0}S_{mn}^j i\kappa_n^j$, and the matrix S^j in the homogeneous dielectric $(j = 1,3)$ is identity matrix $S_{mn}^j = \delta_{mn}$.

The propagations of the amplitude operators in the slab region are determined by the solution of quantum Langevin equations which mean the spatial evolutions of the amplitude operators, the annihilation operators at $z = l/2$ can be written in terms of the annihilation operators at $z = -l/2$ and noise source operator

$$\begin{pmatrix} e^{i\beta^2 l/2}\hat{o}^{2+}(l/2,\omega) \\ e^{-i\beta^2 l/2}\hat{o}^{2-}(l/2,\omega) \end{pmatrix} = \begin{pmatrix} e^{i\kappa^2 l} & 0 \\ 0 & e^{-i\kappa^2 l} \end{pmatrix}\begin{pmatrix} e^{-i\beta^2 l/2}\hat{o}^{2+}(-l/2,\omega) \\ e^{i\beta^2 l/2}\hat{o}^{2-}(-l/2,\omega) \end{pmatrix}$$
$$+\begin{pmatrix} e^{i\kappa^2 l/2}\sqrt{2\gamma^2} & 0 \\ 0 & e^{-i\kappa^2 l/2}\sqrt{2\gamma^2} \end{pmatrix}\begin{pmatrix} \hat{G}^+(\omega) \\ \hat{G}^-(\omega) \end{pmatrix}, \tag{S18}$$

here the elements of the columns $\hat{G}^+(\omega)$ and $\hat{G}^-(\omega)$ associated with the slab read

$$\hat{G}_n^{\pm}(\omega) = \pm i\sqrt{\frac{1}{2\gamma_n^2}}\int_{-l/2}^{l/2} d\tilde{z}e^{\mp i k_n^2\tilde{z}}\sum_m \frac{S_{mn}^{2*}}{2\kappa_n^2}\hat{J}_{my}^2(\tilde{z},\omega), \tag{S19}$$

Based on the relations between amplitude operators at different regions expressed in equations (S16), (S17), (S18) and the definition of $\hat{g}^{\pm}(\omega)$, we can get the input-output relation for the grating in transfer matrix form

$$\begin{pmatrix} \hat{a}^{3+}(l/2,\omega) \\ \hat{a}^{3-}(l/2,\omega) \end{pmatrix} = \begin{pmatrix} T_{11} & T_{12} \\ T_{21} & T_{22} \end{pmatrix}\begin{pmatrix} \hat{a}^{1+}(-l/2,\omega) \\ \hat{a}^{1-}(-l/2,\omega) \end{pmatrix} + \begin{pmatrix} t_{11} & t_{12} \\ t_{21} & t_{22} \end{pmatrix}\begin{pmatrix} \hat{G}^+(\omega) \\ \hat{G}^-(\omega) \end{pmatrix}. \tag{S20}$$

The commutation relations between input photon operators in air and excitations with the slab can be derived based on their definitions

$$\left[\hat{a}_m^{1+}(z,\omega), \hat{a}_n^{1+\dagger}(z',\omega')\right] = e^{-\gamma_m^1|z-z'|}\delta_{mn}\delta(\omega-\omega'), \tag{S21a}$$
$$\left[\hat{a}_m^{3-}(z,\omega), \hat{a}_n^{3-\dagger}(z',\omega')\right] = e^{-\gamma_m^3|z-z'|}\delta_{mn}\delta(\omega-\omega'), \tag{S21b}$$
$$\left[\hat{a}_m^{1+}(z,\omega), \hat{a}_n^{3-\dagger}(z',\omega')\right] = \left[\hat{a}_m^{3-}(z,\omega), \hat{a}_n^{1+\dagger}(z',\omega')\right] = 0, \tag{S21c}$$
$$\left[\hat{a}_m^{1+}(z,\omega), \hat{g}_n^{\pm\dagger}(\omega')\right] = \left[\hat{a}_m^{3-}(z,\omega), \hat{g}_n^{\pm\dagger}(\omega')\right] = 0, \tag{S21d}$$

In this part we obtain the input-output relation in transfer matrix form and the commutation relations between the input operators for the grating.

References

1. Khanbekyan, M., Knöll, L. & Welsch, D.-G. Input-output relations at dispersing and absorbing planar multilayers for the quantized electromagnetic field containing evanescent components. *Phys. Rev. A* **67,** 063812–063823 (2003).
2. Matloob, R. & Loudon, R. Electromagnetic field quantization in absorbing dielectrics. *Phys. Rev. A* **53,** 4567–4582 (1996).
3. Gruner, T. & Welsch, D.-G. Green-function approach to the radiation-field quantization for homogeneous and inhomogeneous kramers-kronig dielectrics. *Phys. Rev. A* **53,** 1818–1829 (1996).
